# Supplementary figures and images for: Activating and inhibiting nucleotide signals coordinate bacterial anti-phage defense
Source: bioRxiv. 2025 Jul 9:2025.07.09.663793. Preprint. [Version 1] doi: 10.1101/2025.07.09.663793 (PMC12265719; doi:10.1101/2025.07.09.663793)

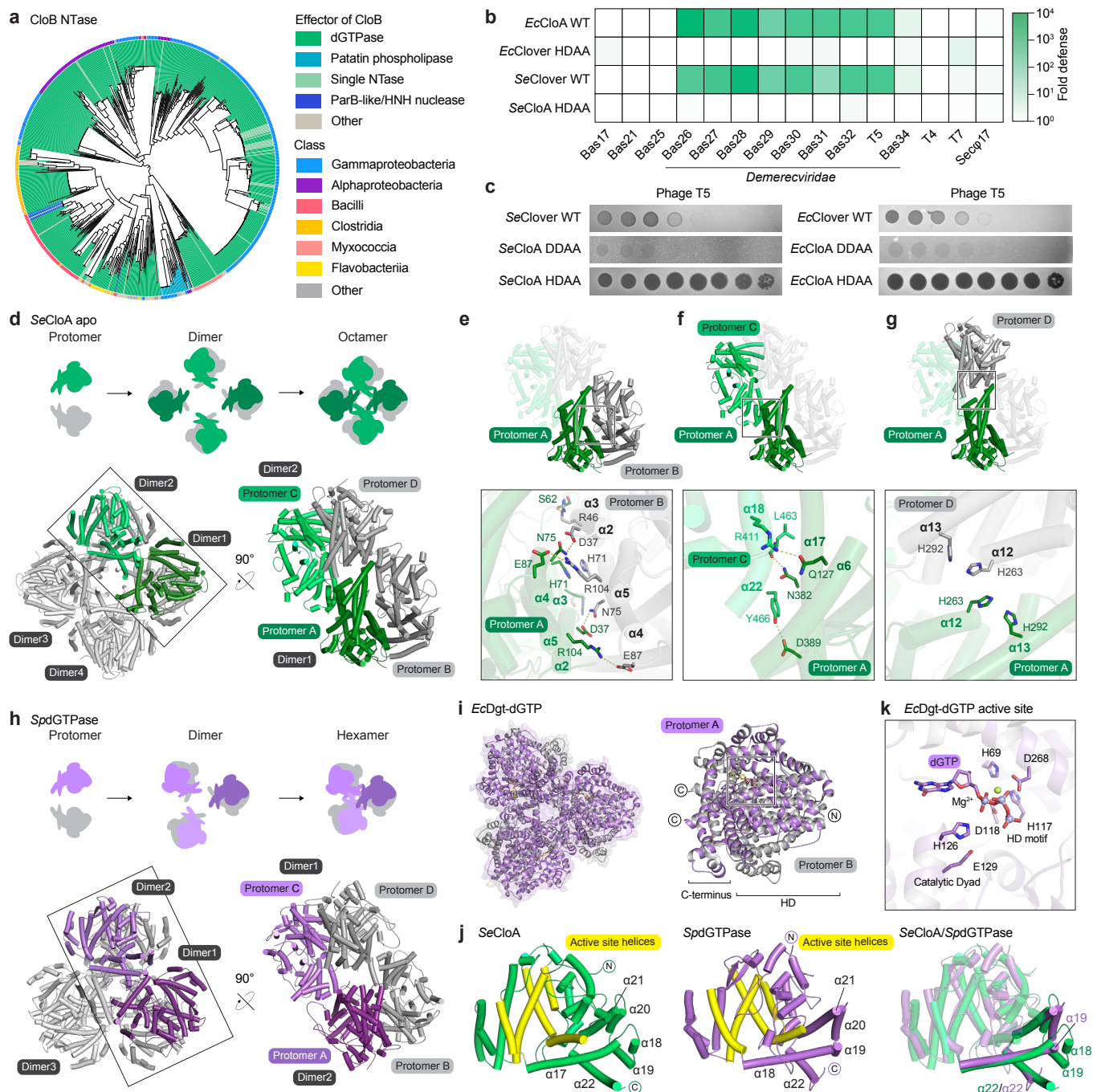

ED Figure 1

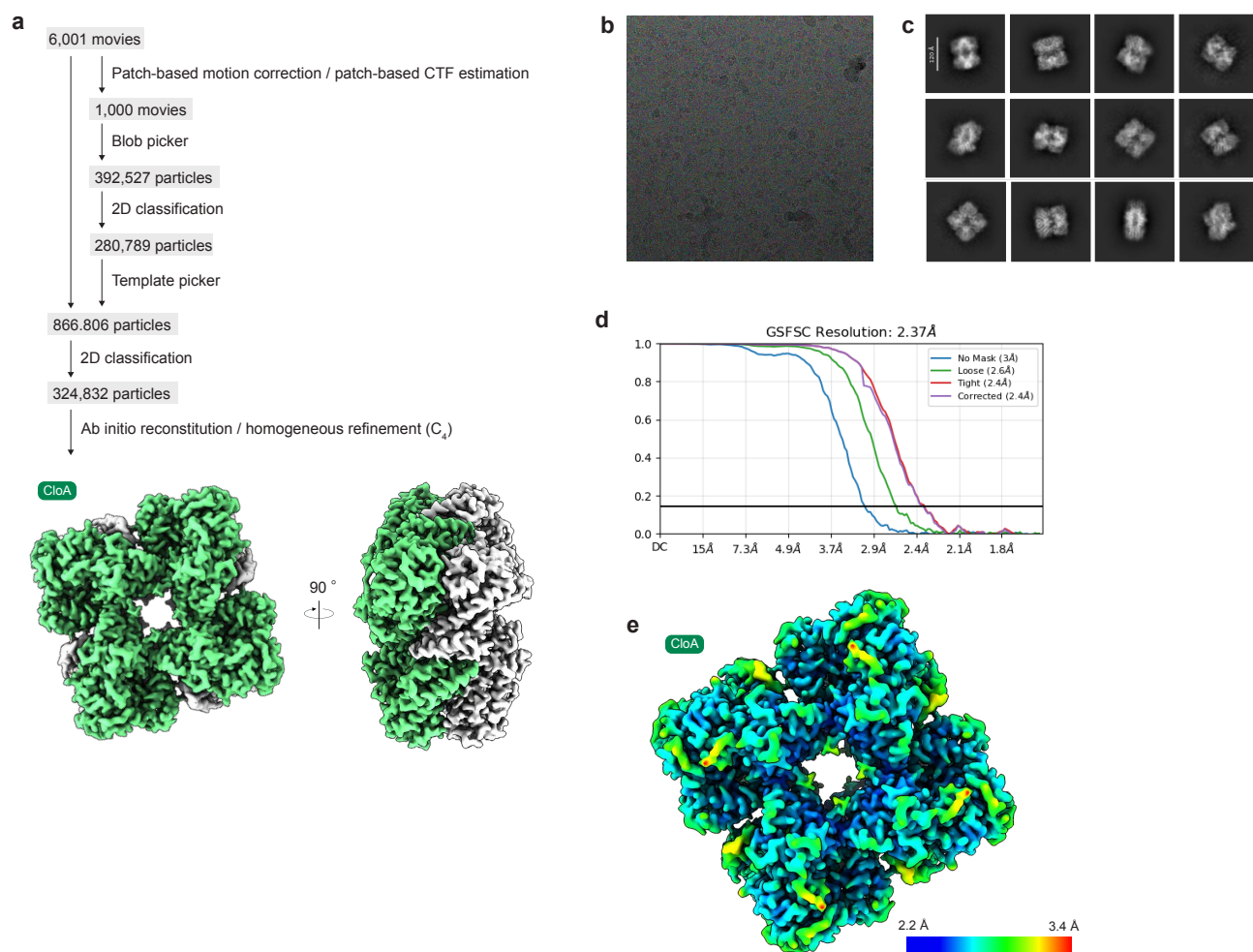

ED Figure 2

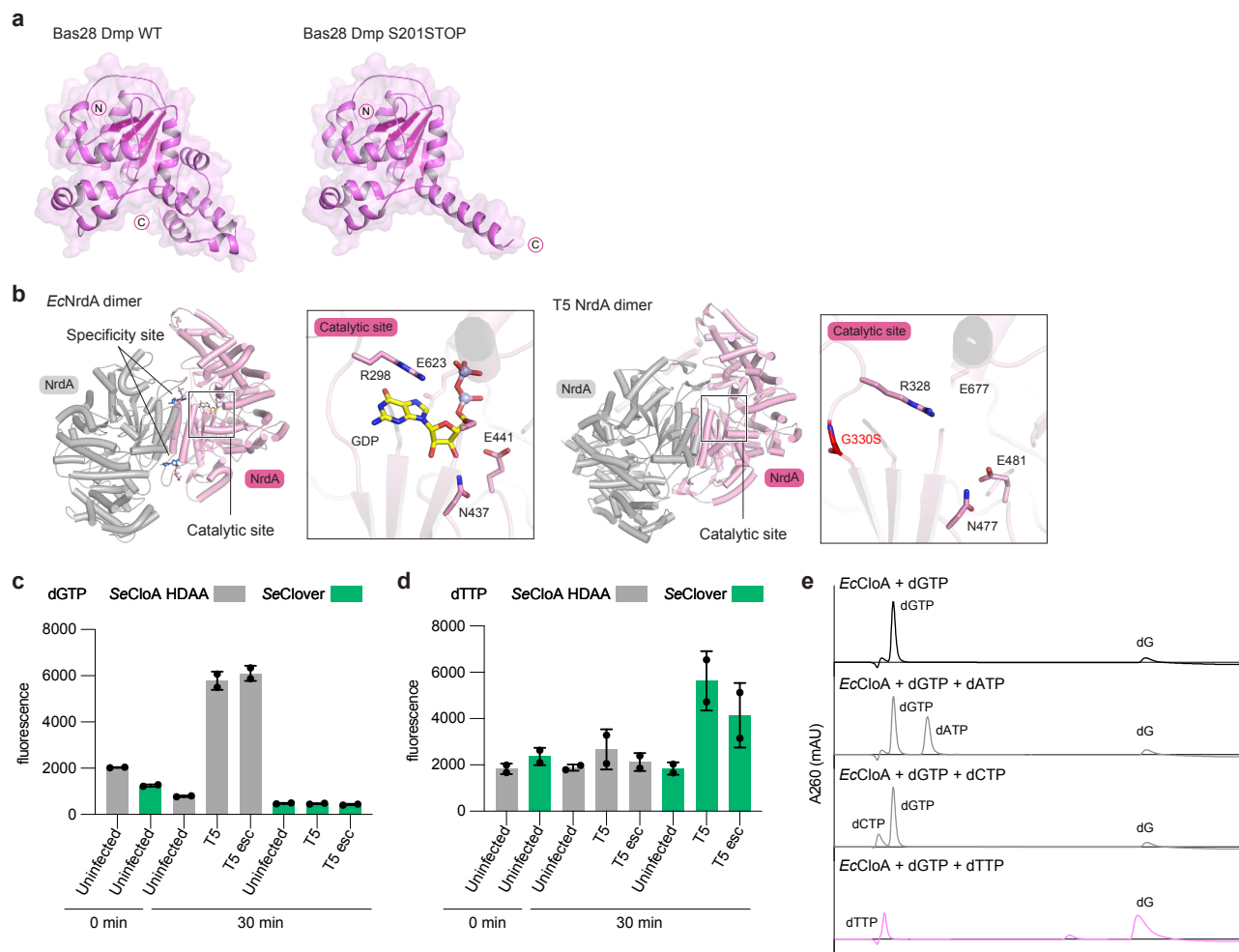

ED Figure 3

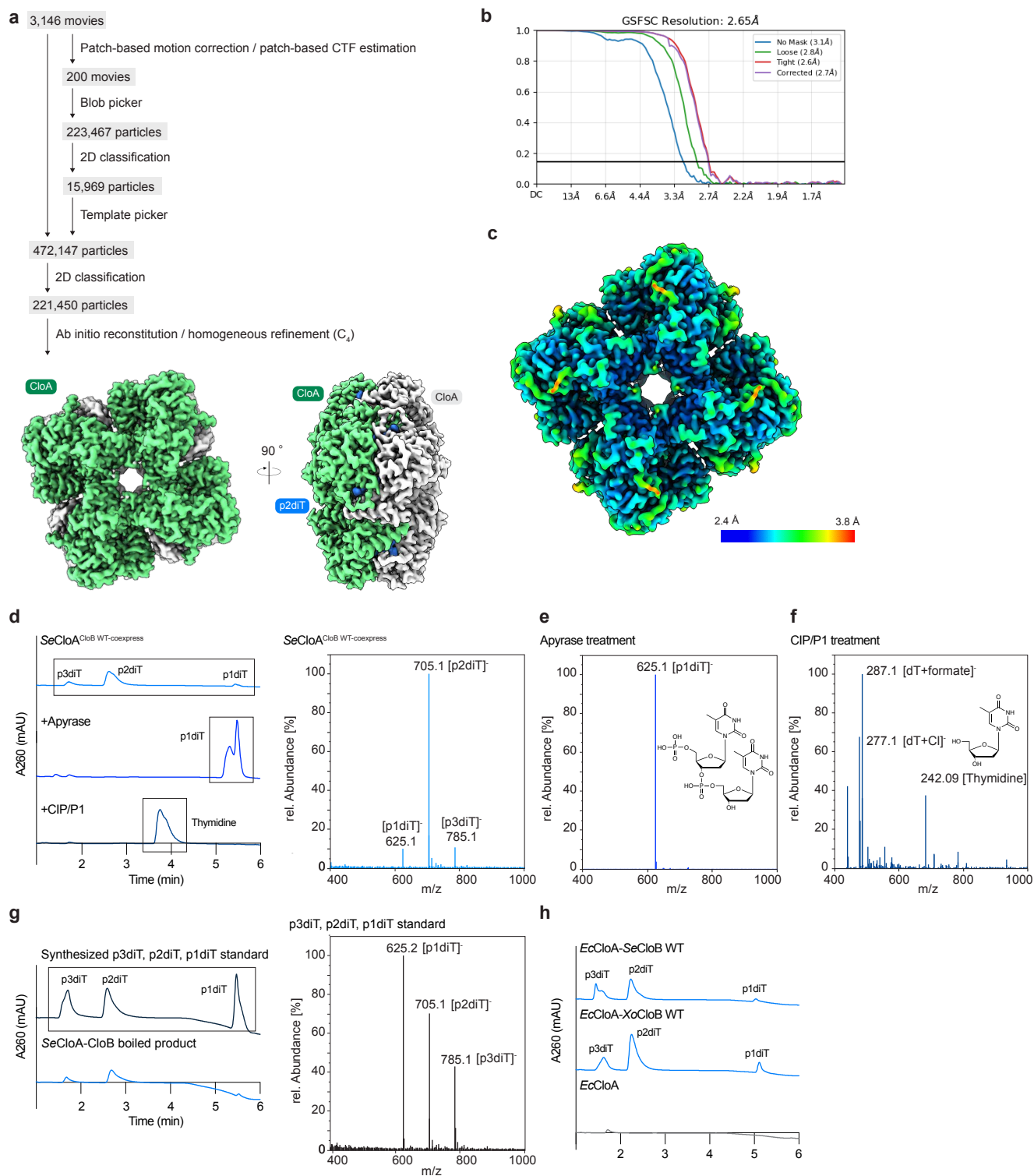

ED Figure 4

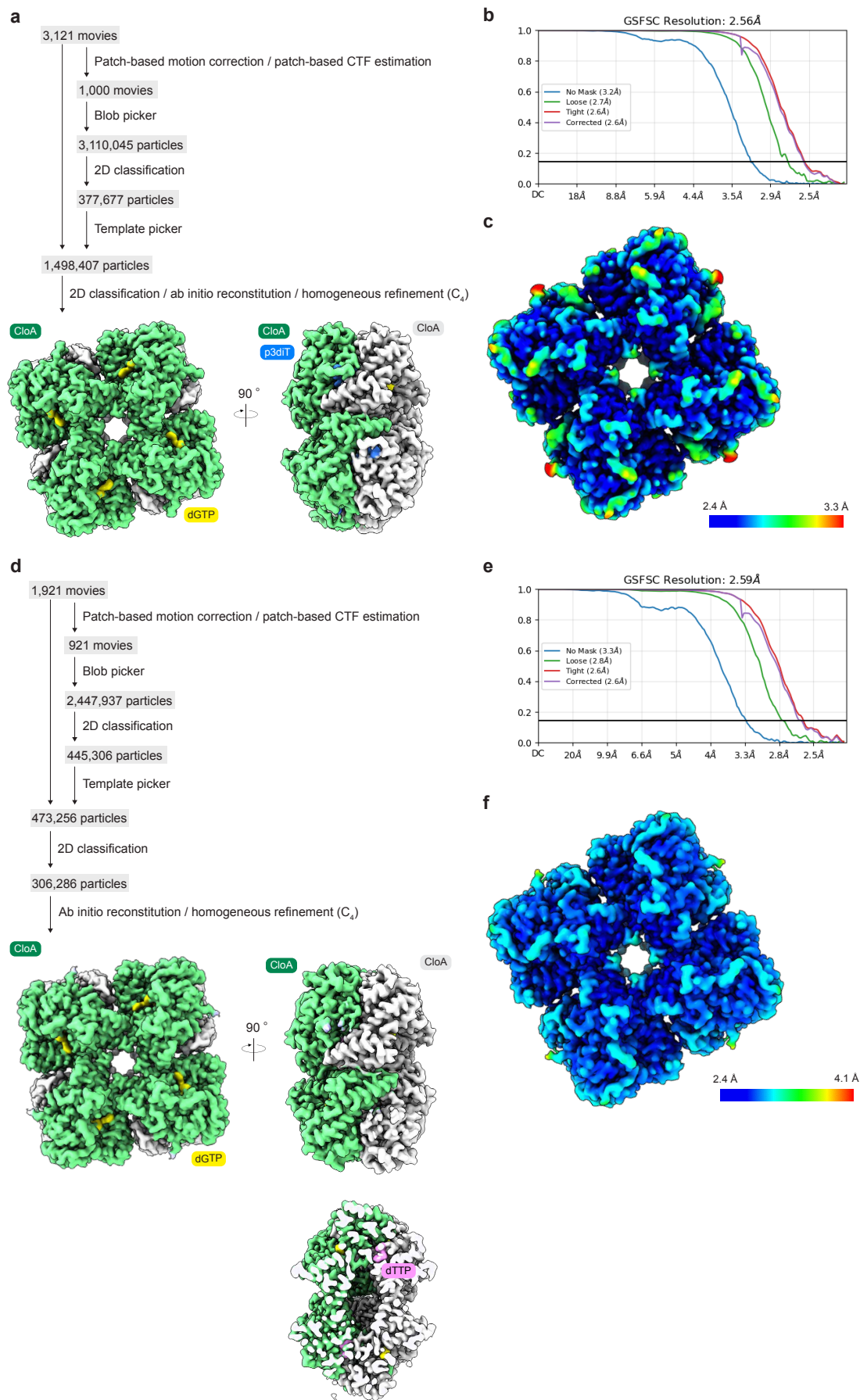

ED Figure 5

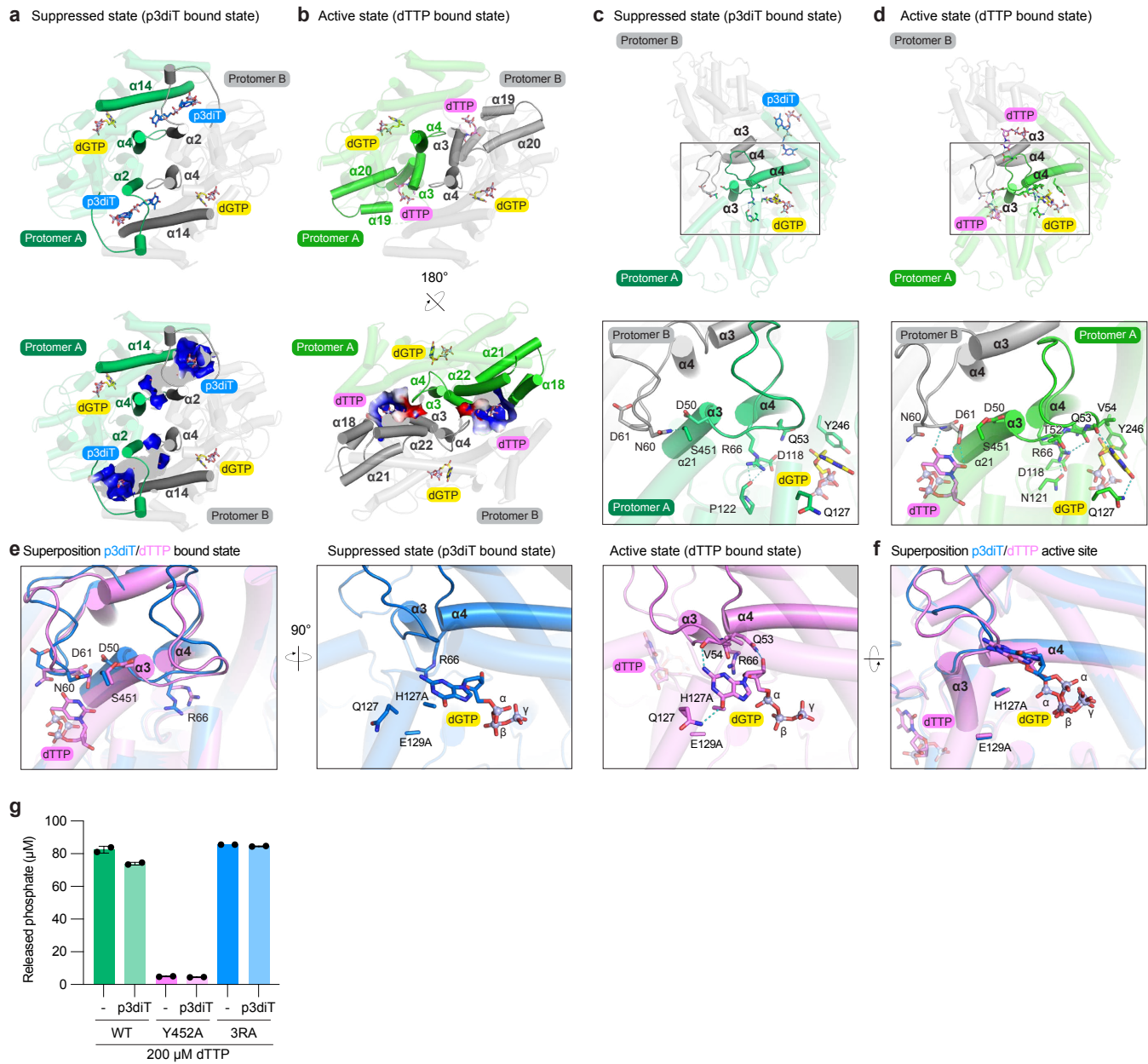

Supplement: Supplement 1 [file media-1.zip › Yamaguchi et al Extended biorxiv (07.09.25).pdf]
